# Effect of Cooling Medium on LDPE Dielectric Properties

**DOI:** 10.3390/polym14030425

**Published:** 2022-01-21

**Authors:** Yujia Cheng, Guang Yu, Zhuohua Duan

**Affiliations:** Mechanical and Electrical Engineering Institute, University of Electronic Science and Technology of China, Zhongshan Institute, Zhongshan 528400, China; Chengyujia1068@163.com (Y.C.); duanzhuohua@163.com (Z.D.)

**Keywords:** cooling medium, LDPE, dielectric properties, space charge properties

## Abstract

Polyethylene, with its excellent mechanical and dielectric properties is used as an insulator for high-voltage direct current (HVDC) transmission. In ultra-high-voltage direct current (UHVDC) transmission, the ageing of insulation materials caused by space charge under high DC voltage becomes serious. Therefore, restraining the space charge and improving the dielectric properties of HVDC cables is important. In this study, low-density polyethylene (LDPE) was used as the raw material and combined with cooling media in a vulcanizing press. A polarizing microscope was used to observe the samples’ crystal morphologies. The space charge accumulation and dispersion were detected using pulsed electro-acoustic. Additionally, dielectric properties such as electrical conductivity and dielectric frequency spectrum were tested. The grain size in the air-cooled LDPE samples was found to be large and unevenly dispersed. However, the grain sizes in the water and oil cooling LDPE samples were small. The mean charge density of the oil cooling samples was the lowest. Under a short circuit measurement, more space charges were found in the natural and rapid air cooling samples. The mean charge densities of these two samples were high, with a fast decay rate. With an 8 and 50 kV/mm electric field strength, the oil cooling samples’ conductivity was the highest and lowest, respectively.

## 1. Introduction

With the development of industry, there must be higher standards for the transmission capacity and operational stability of power systems [1]. Energy resource distribution and electrical load are imbalanced in different areas. Therefore, the long-range transmission of electrical energy is required, which introduces HVDC. Compared with traditional high-voltage AC transmission (HVAC), HVDC possesses several advantages, such as low cost, short line corridor, low electric energy loss, fast power adjustment, and reliable operation. In addition, a thyristor inverter is used to rapidly adjust the active power of the DC line in the AC/DC hybrid transmission system [2,3,4,5,6]. The power flow direction is changed, which improves the system reliability. In traditional long-distance HVDC systems, oil-filled power cables and oil paper cables are commonly used [7,8]. However, the use of these cables cause problems, such as oil leakage, rendering them inconvenient in use. Plastic cables possess some advantages, such as a large transmission capacity, small mass, and long-term operational stability. Thus, these cables are widely used in AC transmission systems [9,10,11]. Plastic cables are currently used in DC transmission systems. Among them, polyethylene possesses excellent dielectric and mechanical properties and is widely used for power cable insulation [12,13,14]. However, owing to the effects of a long-term higher electric field, space charges accumulate, which degrade the polyethylene insulation properties [15,16]. Moreover, material ageing will result in insulation failure. To improve the service life of insulation materials and the reliability of equipment operation, many researchers have attempted to change the insulation material preparation process, to improve the dielectric properties of the materials. In addition, in polyethylene sample preparation, all samples must undergo a cooling process [17,18]. The different cooling processes have different effects on the crystallization behavior of polyethylene samples. The crystal size and integrity affect the sample electrical conductivity characteristics [19,20]. The space charge generation, motion, and attenuation are, not only related to the applied voltage, but also related to a sample’s crystalline state [21]. Therefore, during sample preparation, the effects of different cooling processes on the dielectric properties of polyethylene samples need to be explored.

In this study, different polyethylene insulation materials were prepared by air natural cooling, air rapid cooling, water cooling, and oil cooling. Based on pulsed electro-acoustic (PEA), the different sample space charge accumulations and decays under pressure and short circuit were tested. The effect of different cooling mediums on the polyethylene space charge behavior was explored. In addition, a polarization microscope (PLM) was used to observe the crystalline morphology of polyethylene. The effect of different cooling media on the crystalline morphology of the materials was analyzed. A pico-ammeter was used to establish the volt–ampere characteristics of the test equipment, from which the relationship between the conductivity of the different samples and the field strength could be determined. The effect of different cooling mediums on the material conductance was analyzed. Moreover, the different polyethylene samples were subjected to a dielectric spectrum test, from which the relationship between the test frequency, the dielectric constant, and dielectric loss factor was recorded. Finally, the effect of different cooling media on the dielectric constant and dielectric loss factor was explored.

## 2. Polymer Preparation Process and Crystalline Morphology Analysis

To explore the effect of the different cooling mediums on polyethylene space charges and other dielectric properties, the polyethylene samples were melt-pressed by a vulcanizing press for 15 min. The temperature was 150 °C, and the pressure was 15 MPa. Subsequently, all the samples were cooled down to room temperature using different cooling media. Samples of different thicknesses were prepared. The sample number, thickness, cooling medium, and practical applications are listed in Table 1.

In this study, the samples were subjected to natural air cooling, rapid air cooling, water cooling, and oil cooling. Natural air cooling means that the samples were melt-pressed in a vulcanizing press for 15 min. These samples were then removed and exposed to air for natural cooling. In rapid air cooling the samples were cooled rapidly by an electric fan after being removed from the vulcanizing press. Water cooling means that the samples were cooled rapidly in warm water after being removed from the vulcanizing press. The samples were then dried in air. Oil cooling means that the samples were cooled rapidly in cable oil after being removed from the vulcanizing press and then dried in air.

**Figure 1:**
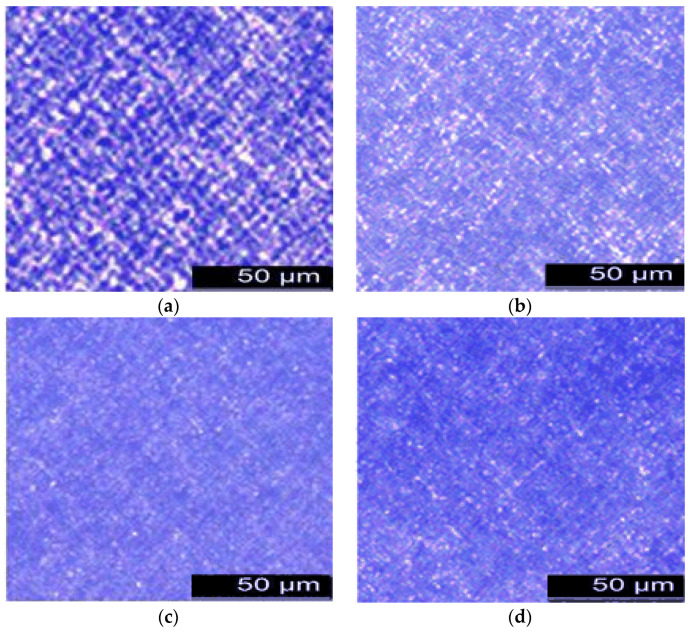
PLM images of different samples. (**a**) PLM image of sample 1. (**b**) PLM image of sample 2. (**c**) PLM image of sample 3. (**d**) PLM image of sample 4.

The crystalline state is an important aggregation state of polymers. Extensive experimental results have demonstrated that the aggregation of high polymer chains will form crystals, as long as the molecular chains possess the necessary regularity and the conditions (such as temperature and time) are suitable. Several polymers, such as polyethylene, polypropylene, and polystyrene, possess crystallinity. Therefore, in this study, polyethylene was tested using PLM. The different polymer crystal morphologies were related to the crystal conditions. Crystal platelets are commonly folded chain lamellae and straight-chain lamellae. The morphology of the polyethylene crystals under different conditions is shown in Figure 1.

The polymer crystals show the birefringence property, thus, there are light and dark areas in the polarized light interference crystallization images. PLM was used to observe the crystalline morphology of polyethylene. Figure 1a shows the crystalline morphology of sample 1. As observed, the crystalline grains were large and dispersed unevenly. This is because a low cooling rate prolongs the crystal growth time. The crystal nucleus creates less crystals, and the grain size is large. Figure 1b shows the crystalline morphology of Sample 2. Compared with Figure 1a, the grains are small and dispersed evenly. This is because a high cooling rate decreases the time of crystal growth. The crystal nucleus creates more crystals, and the grain size is small. Figure 1c,d show the crystalline morphologies of samples 3 and 4, respectively. The grains were smaller and more evenly dispersed. This illustrates that the cooling medium has a significant effect on the crystalline morphology of polyethylene. In a previous study on the effect of a nucleating agent on high-density polyethylene (HDPE) properties, the crystalline morphology in different kinds of samples showed significant differences. The grains were larger in HDPE samples with natural air cooling, and the grains were small in HDPE samples with rapid air cooling. This is consistent with the conclusions of this study. Moreover, the change of crystalline morphology affects the trap depth and density of samples, which affect the space charge distribution. The sample dielectric properties are also changed.

In order to explore the different sample crystallizations, a DSC-1 (produced by Mettler, Toledo, Spain) was used to test the parameter changes of different samples during crystallization. The PLM test results were further verified using a DSC test. The effect of cooling rate and medium on LDPE crystalline morphology were explored. Furthermore, the effect on sample space charges distribution could be analyzed. This experiment was carried out under nitrogen protection. The nitrogen flow rate was 150 mL/min. The heating rate in this experiment was 10 °C/min.

**Figure 2:**
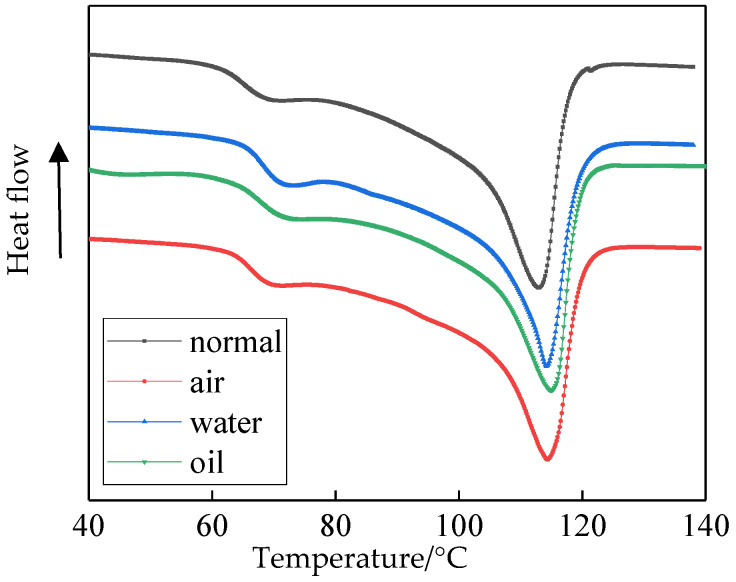
Heating curve of different samples.

The DSC rising melting curves of four samples are shown in Figure 2. *θ_m_* is the melting temperature, which is the melting peak temperature. This can reflect the heat resistance of the polymers. Δ*H_m_* is the melting enthalpy (J/g), which can be calculated by the areas surrounding the baseline of the melting peak. This can reflect the motility of the polymer molecular chains. *X_c_* is crystallinity, which can be calculated by the percentage of 100% crystallization LDPE melting enthalpy, *H_N_* and Δ*H_m_*. This can characterize the crystalline proportion in a semi-crystalline polymer. *X_c_* can be calculated by Equation (1). *H_N_* is 293.6 J/g (experimental instrument calibration). *X_c_* and *θ_m_* are listed in Table 1.

*X*_c_ = Δ*H_m_*/*H_N_* × 100% (1)

From Figure 2 and Table 1, the *X_c_* and *θ_m_* of sample1 are the least. Therefore, the crystallinity of sample 1 is low, and the heat resistant is poor. The crystallinity of the other three samples increased. *θ_m_* will move to a high-temperature zone. Among them, the material crystallinity in sample 4 increased drastically. The material crystallinity in sample 3 was slightly higher than in sample1. The *X_c_* and *θ_m_* of sample 2 were both between those of sample 3 and sample 4. This further verifies the PLM test result. The cooling mediums had a great effect on the polyethylene crystalline morphology. Among them, the crystal size was large and crystallinity low in sample 1. While, the heat resistance was strong and the crystallinity high in sample 4.

## 3. Effect of Cooling Medium on Material Space Charges

Space charge generation, migration, and dissipation change the internal electric field of insulation materials, which severely disrupts the service life of electrical equipment. Different cooling mediums can change the polymer microstructure, which also affects the macroscopic electrical properties. In this section, the effect of the cooling media on space charges is analyzed.

During the preparation of polyethylene samples, impurities are introduced into the samples, which causes more traps. The energy level of these traps is between the conduction and valence bands. Space charges are moving charges, which can be captured by the traps. The four samples were subjected to high-pressure and short-circuit tests that changed the crystalline morphology of the materials. Therefore, the trap depth was reduced. Moreover, the charge mobility and dissipation rate increased.

**Figure 3:**
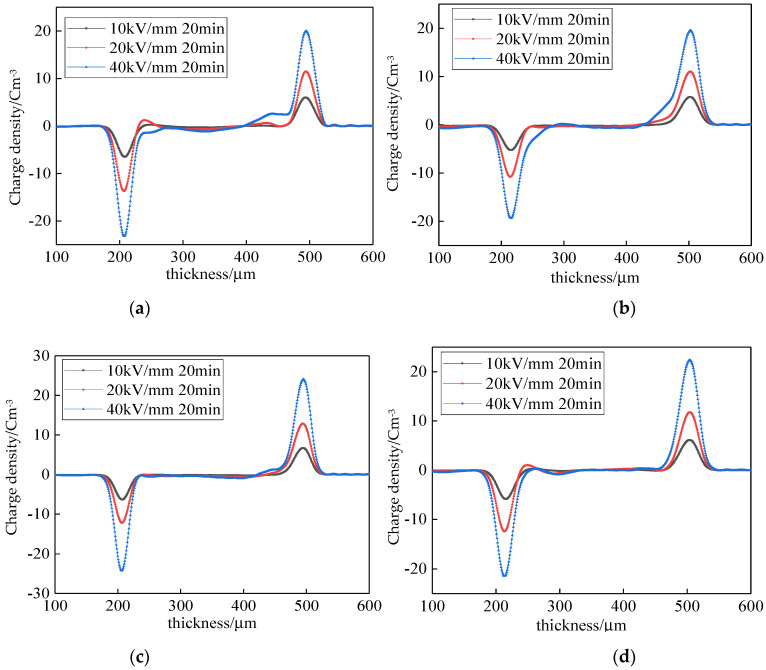
Space charge accumulation of different samples under an electric field. (**a**) Space charge accumulation of sample 1; (**b**) Space charge accumulation of sample 2. (**c**) Space charge accumulation of sample 3; (**d**) Space charge accumulation of sample 4.

Space charge accumulation characteristics under high pressure: The space charge distributions of the LDPE with different cooling media under high pressure are shown in Figure 3. The space charge distribution of sample 1 is shown in Figure 3a. Under a 20 kV/mm electric field, heteropolarity charges appear around the negative electrode, with a peak value of 1.2425 C/m^3^. This is due to the decomposition of impurities to some charges under high pressure. These charges are captured by the traps during the migration process, and heteropolarity space charges appear. Under a 40 kV/mm electric field, a large number of homopolar charges were found around the electrodes, with peak values of 1.284 and 2.5373 C/m^3^. Moreover, only a small amount of negative charge, with a peak value of 1.2143 C/m^3^, was found in the samples. These charges were produced by electrode injection.

The space charge distribution of sample 2 under high pressure is shown in Figure 3b. Compared with the samples of natural air cooling, heteropolar charges do not appear in the negative electrode under the 20 kV/mm electric field. Under a 40 kV/mm electric field, more charges are injected by the electrodes. The negative charge in the sample is 0.6436 C/m^3^, which is 46.99% lower than that of sample 1. This is because the rapid air cooling changes the crystalline morphology of LDPE. The grain size decreases, and the trap depth become shallow. The space charges transfer easily, and, thus, the accumulation of space charges decreased. Under a 40 kV/mm electric field, the charges injected by the electrodes increased. This is because rapid air cooling changed the surface morphology of the samples, which caused the work function between the electrodes to change the surface of the samples.

The space charge distribution of sample 3 under high pressure is shown in Figure 3c. Compared with sample 1, the charges injected by the electrodes were significantly inhibited. There were no heteropolar charges in the negative electrode. Under a 40 kV/mm electric field, there were few charges injected by the positive electrodes. There were few negative charges in this sample. The peak value was 0.8351 C/m^3^, which is 31.2% lower than that of sample 1. The space charge distribution of sample 4 under high pressure is shown in Figure 3d. Compared with sample 1, there were no charges injected by the electrodes. Under a 20 kV/mm electric field, the heteropolar charges around the negative electrode and the sample charges were lower. Compared with sample 2, the charges injected by the electrodes were further inhibited. This is because water cooling and oil cooling significantly affected the LDPE crystalline morphology. The grain size decreased further with a shallow trap depth. The space charge transition was easy. The changes in the crystalline morphology of the samples affected the surface morphology of the samples, which inhibited the charges injected by the electrodes. In addition, the surface morphology of the samples was affected by water cooling and oil cooling, which changed the work function between the electrodes and the sample surface. This also decreased the charge injected by the electrodes.

To characterize the sample space charge accumulation under high pressure, the mean charge density $Q_{\mathrm{med}}$ was introduced. It can be calculated using Equation (2).

(2) $$Q_{\mathrm{med}}=\frac{1}{x_{2}-x_{1}}\int_{x_{1}}^{x_{2}}\left|\rho (x)\right|dx$$

In Equation (2), $x_{1}$ and $x_{2}$ are the end of the cathodic peak and the start of the positive peak, respectively. ρ(x) is the space charge density under short-circuit.

**Figure 4:**
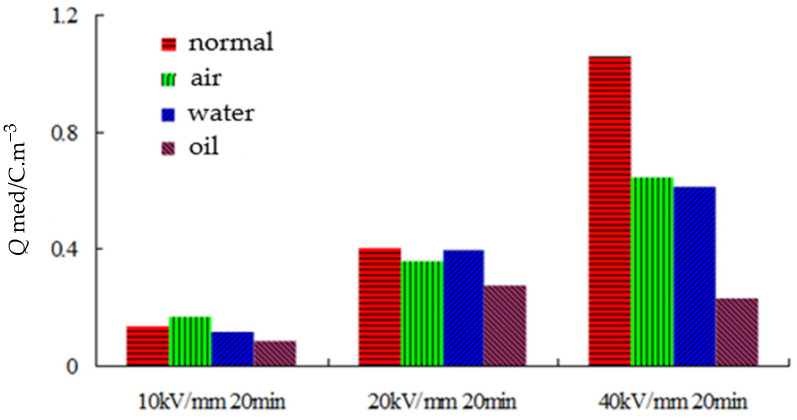
Mean charge density of different samples under various electric fields.

Under high pressure, the mean charge densities of the different samples are shown in Figure 4. Under a 10 kV/mm electric field, the mean charge density of sample 2 was the highest, while that of sample 4 was the lowest. The former is 1.92 times more than the latter. Under 20 kV/mm and 40 kV/mm electric fields, the mean charge density of sample 1 was the highest, while that of sample 4 was the lowest. The former are 1.48 and 4.58 times more than the latter under the two electric fields. This illustrates that the cooling medium inhibits space charges under high pressure. Among them, the space charge inhibition effect of oil cooling was the best.

**Figure 5:**
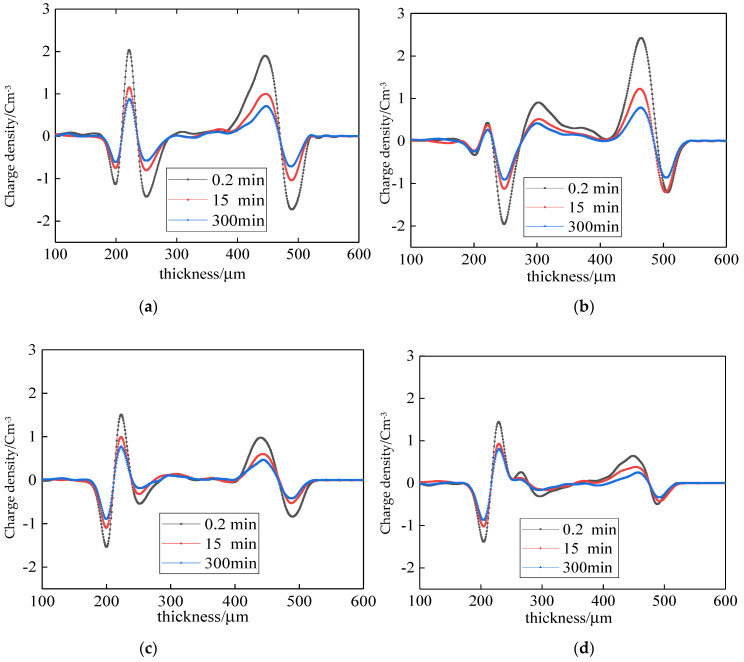
Space charge dispersion of different samples under short-circuit. (**a**) air normal cooling; (**b**) air rapid cooling. (**c**) water cooling; (**d**) oil cooling.

Space charge attenuation characteristics under short circuit:

Under a short circuit, the space charge dispersion of different samples is shown in Figure 5. For a quantitative comparison of space charges in different samples, the peak values of space charges in different sample positions were recorded, as shown in Table 2. The space charge dispersion of sample 1 under short circuit is shown in Figure 5a. More space charges remained in 0.2 min. The heteropolar charges were around the negative electrode, with a peak value of 2.0396 C/m^3^. The homopolar charges were around the positive electrode, with a peak value of 1.9007 C/m^3^. There were plenty of negative charges in the samples, with a peak value of 1.4248 C/m^3^. The space charges gradually disappeared with time. There were a few remaining space charges at 300 min. The heteropolar charge around the negative electrode decreased to 0.8799 C/m^3^. The homopolar charge around the positive electrode decreased to 0.7034 C/m^3^. The negative charge in this sample decreased to 0.5782 C/m^3^. This is because the trap depth was deeper, and the density is high in sample 1. More charges were injected by the electrodes. Thus, more space charges remain at 0.2 min and 300 min. As time passes, the space charges captured by shallow traps are detrapped, which decreases the space charges.

The space charge decay of sample 2 under short circuit is shown in Figure 5b. Compared with sample 1, the space charge distribution was different. The homopolar charges were in the negative electrode, and the positive charges were in the samples. The charges around the negative electrode decreased by 4.15% at 0.2 min. The charge peak value around the positive electrode increased by 27.5% and decreased by 36.4% in the sample. At 300 min, the charge peak values in the negative and positive electrodes increased by 3.84% and 10.9%, respectively. Moreover, the charge peak value in the sample decreased by 29.7%. This illustrates that rapid air cooling changes the crystalline morphology of LDPE. Furthermore, the space charge attenuation was affected. The decrease in grain size caused the trap depth to become shallow. Space charge transfer was easy, and the remaining space charges decreased.

The space charge attenuation of sample 3 under short-circuit is shown in Figure 5c. The space charge distribution was similar to that of sample 1. The charge peak values around the negative electrode and positive electrode at 0.2 min decreased by 26.06% and 48.62%, respectively. The charge peak value in the sample decreased by 62.09%. The charge peak values around the negative and positive electrodes at 300 min decreased by 12.89% and 33.7%, respectively. The charge peak value in the sample decreased by 67.9%. The remaining space charges evidently decreased compared with those of sample 2. The space charge attenuation of sample 4 under short circuit is shown in Figure 5d. The space charge distribution of sample 4 was similar to that of sample 1. The remaining space charges were lowest at 0.2 min. The charge peak values around the negative electrode and positive electrode decreased by 29.1% and 65.99%, respectively. The charge peak value in the sample decreased by 78.04%. The least remaining space charges were at 300 min The charge peak values around the negative electrode and positive electrode decreased by 8.51% and 64.74%, respectively. The charge peak value in the sample decreased by 71.23%. Compared with sample 2, the charge peak values of the sample and positive electrode in sample 4 were clearly decreased. This illustrates that the space charge attenuation in samples 3 and 4 was higher. The remaining space charges were smaller. This is due to the water and oil cooling, which further decreased the grain size. The trap depth was shallow, and the space charge transition was easy.

To characterize the rate of space charge attenuation under short circuit, the attenuation rate of the mean charge density $V_{Q\mathrm{med}}$ was introduced, which can be calculated by Equation (3).

(3) $$V_{Q\mathrm{med}}=\frac{\Delta \bar{Q_{l}}}{\Delta t}=\frac{\left|\bar{Q_{l2}}-\bar{Q_{l1}}\right|}{t_{2}-t_{1}}$$

In Equation (3), $\bar{Q_{l1}}$ and $\bar{Q_{l2}}$ are the sample mean charge densities in $t_{1}$ and $t_{2}$.

**Figure 6:**
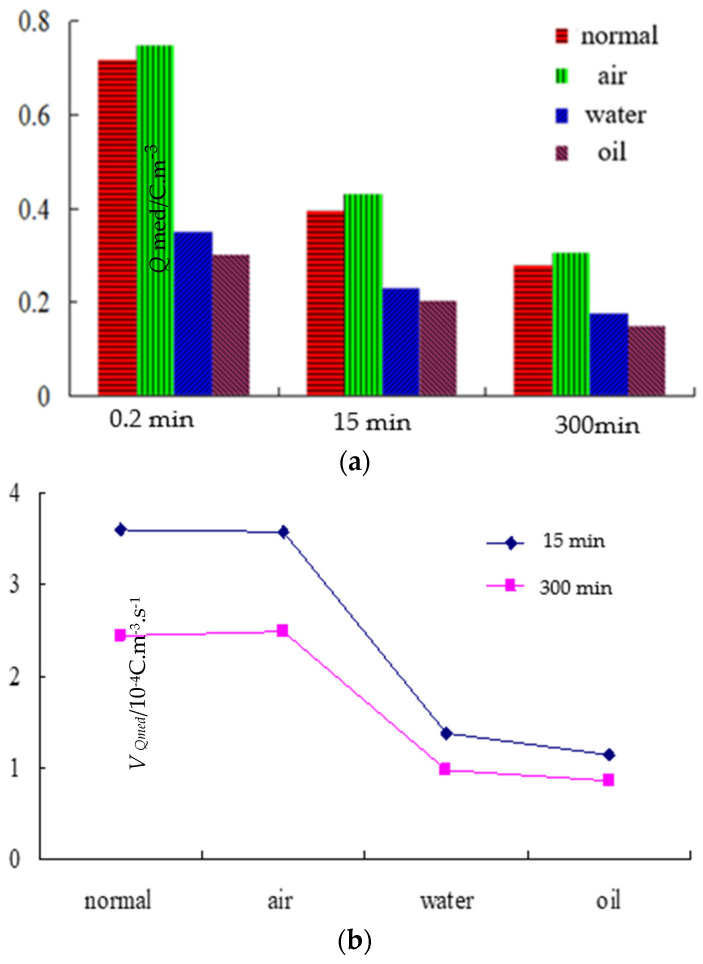
Mean charge density and mean charge attenuation rate of different samples under short circuit. (**a**) Mean charge density of different samples. (**b**) Mean charge attenuation rate of different samples.

The mean charge density and attenuation rate of different samples in short circuit are shown in Figure 6. From Figure 6a, at 0.2 min, 15 min, and 300 min, the order of mean charge density of the four samples was: sample 2 > sample 1 > sample 3 > sample 4. At 10 s, the mean charge density of sample 2 was 2.45 times more than that of sample 4. From Figure 6b, at 900 s and 1800 s, the mean charge density and attenuation rate of samples 1 and 2 are higher than those of samples 3 and 4. The attenuation rate of sample 2 was 2.6 times more than that of sample 4.

The space charge distribution is related, not only to the cooling medium and nanoparticles, but also to the field strength and pressure time. In this study, sample 1 was used as an example to explore the effect of pressure time on space charges.

**Figure 7:**
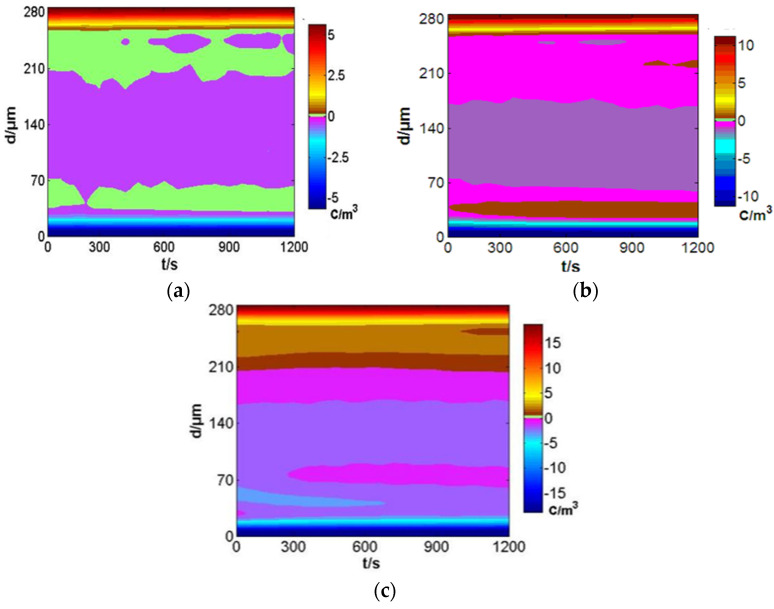
Variation of space charge with time under different electric fields. (**a**) 10 kV/mm. (**b**) 20 kV/mm. (**c**) 40 kV/mm.

The relation between the space charges in sample 1 with time is shown in Figure 7. The x-axis represents the time of the applied voltage. The y-axis represents the sample thickness. The side of the sample that touched the negative electrode was zero. Different space charges are shown in different colors. Under a 10 kV/mm field strength, several negative charges were in the middle of the samples, the amplitude of which was low. With the 245 μm sample thickness, a dynamic change in negative charges trapped and detrapped appeared at 450 s and 1200 s. Under a 20 kV/mm field strength, there were small amounts of heteropolar charges in the 35 μm sample thickness. With increasing time, these charges spread to both sides. With the 240 μm sample thickness, a dynamic change in negative charges trapped and detrapped appeared in 500 s and 900 s. At 1000 s, there were small amounts of positive charges with the 215 μm sample thickness. Under a 40 kV/mm field strength, injected electrons were found in the 65 and 70 μm sample thickness. As time increased, the number of charges decreased. Electron hole injection was found at 210 μm sample thickness. The number remained invariable with time. At 300 s, the negative charges decreased slightly for the 74 μm sample thickness.

Based on the above analysis, the space charge change was complicated with a change in pressurization time. The space charges in most of the sample area remained unchanged. In a few areas, the space charges gradually decreased. In very few areas, space charges changed dynamically.

In summary, after a 40 kV/mm field strength was applied for 20 min, the space charge density in sample 2 was the highest. The space charge density in sample 4 was the lowest. After a 30 min short circuit test, the order of the residual space charges in the four samples was as follows: sample 1 > sample 2 > sample 3 > sample 4. This illustrates that sample 4 could restrain the space charges well.

## 4. Effect of Different Cooling Media on Material Conductivity and Dielectric Properties

The conductivity, dielectric constant, and dielectric loss of the samples were tested, to explore the effect of different cooling mediums on the dielectric properties of LDPE. 

Conductive means the electric current passes through the polymers under a high field. Crystalline and amorphous phases coexist in the polymers. This complex microstructure causes the polymer conductivity to become complicated. In this study, a picoammeter was used to test the conductivity of the different samples. The sample thickness was approximately 200 μm. A vacuum coater was used to plate a three-electrode system onto the sample surface. The conductivity could be calculated using Equation (4).

(4) $$\sigma =\frac{I}{U}\times \frac{4d}{\pi (D_{1}\times g)}$$

In Equation (4), *σ* is the conductivity. *U* is the test voltage. *D* is the sample thickness. *D*_1_ is the inner electrode diameter of the sample surface. *g* is the space length, which was 1 mm.

**Figure 8:**
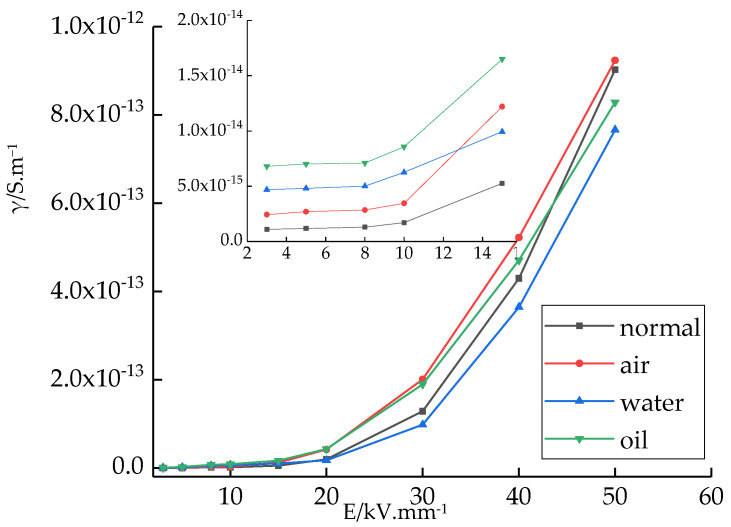
Conductivity variation of different samples under different electric fields.

The variations in the conductivity of the different samples with field strength changes are shown in Figure 8. Under different field strengths, the variation trends of the conductivities of the four samples were different. For a low field strength (lower than 10 kV/mm), the conductivity was lower and changed slightly on increasing the field strength. Under a high field strength (higher than 10 kV/mm), the conductivity increased with the field strength. This is because both crystalline and amorphous areas existed in the LDPE. The structure traps between the crystalline and amorphous areas have a certain effect on conductivity, which is called the space charge limiting current effect. For a low field strength, the charges do not fill the traps. The moving carriers could be captured by the traps, which decreased the carrier concentration. Therefore, the conductivity was lower. As the field strength increased, the charges injected by the electrodes increased significantly. Once the traps were filled by the charges, the carriers were not captured by the traps. As the field strength increased, the carrier concentration increased significantly, and, thus, the conductivity was high.

**Figure 9:**
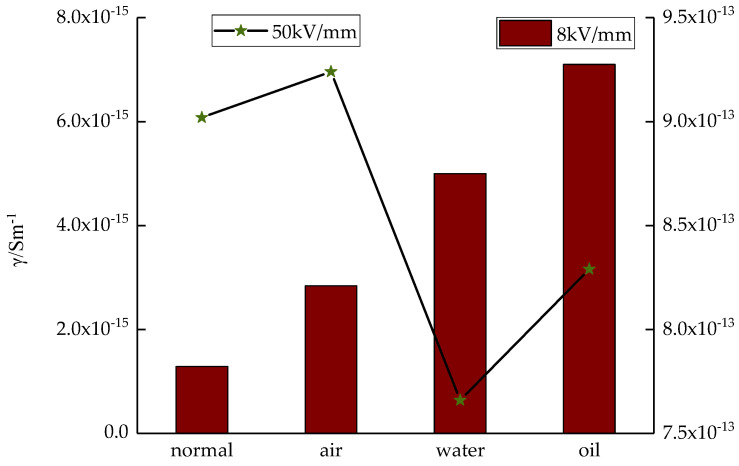
Different sample conductivity under low and high electric fields.

The different cooling media affected the LDPE conductivity, and the changes in conductivity were different under low field and high field strengths. For a better comparison, a sample conductivity line chart under the 8 kV/mm field strength and bar chart under the 50 kV/mm field strength were drawn, as shown in Figure 9. Under an 8 kV/mm field strength, the conductivities of the four kinds of samples were significantly different. The conductivity of sample 1 was the lowest. The conductivity of sample 4 was the highest. It was 5.5 times higher than that of sample 1. Under a 50 kV/mm field strength, the conductivity of the different samples was similar. The order of the conductivity of the four samples was as follows: sample 1 > sample 2 > sample 3 > sample 4.

Under a low field strength, the charges could not be injected into the LDPE samples by the electrodes. The carriers that participate in the conduction process are mainly ions, resulting from ionic conduction. As more impure ions were introduced in the preparation of samples 2, 3, and 4, the carrier concentration increased. In addition, the conductivity was higher. Under a high field strength, more electrons and electron holes were injected by the electrodes. The carriers that participated in the conduction process were electrons, electron holes, and impurity ions. Among these, electrons played the primary role in electronic conduction. As a large number of charges were injected by the electrodes, the carrier concentration increased. The conductivities of the different samples were similar. Among them, the conductivities of samples 3 and 4 were relatively low. This was due to the effect of the cooling media, which changed the crystalline morphology of LDPE. The grain size decreased; therefore, the structure traps between the crystalline and amorphous areas increased. More traps captured more carriers, thereby decreasing the carrier mobility. Therefore, the conductivity decreased.

In summary, under the 50 kV/mm field strength, the conductivity of samples 3 and 4 was lower.

Under an electric field, dielectrics implies that polarization will occur in these materials. Atoms and ions will cause elastic displacement, from which the dipole moment is induced. This is atom displacement or ion displacement polarization. In a dielectric, the natural dipole moment of a polar molecule turns along the electric field, which causes a macroscopic dipole moment. This is the orientation polarization.

**Figure 10:**
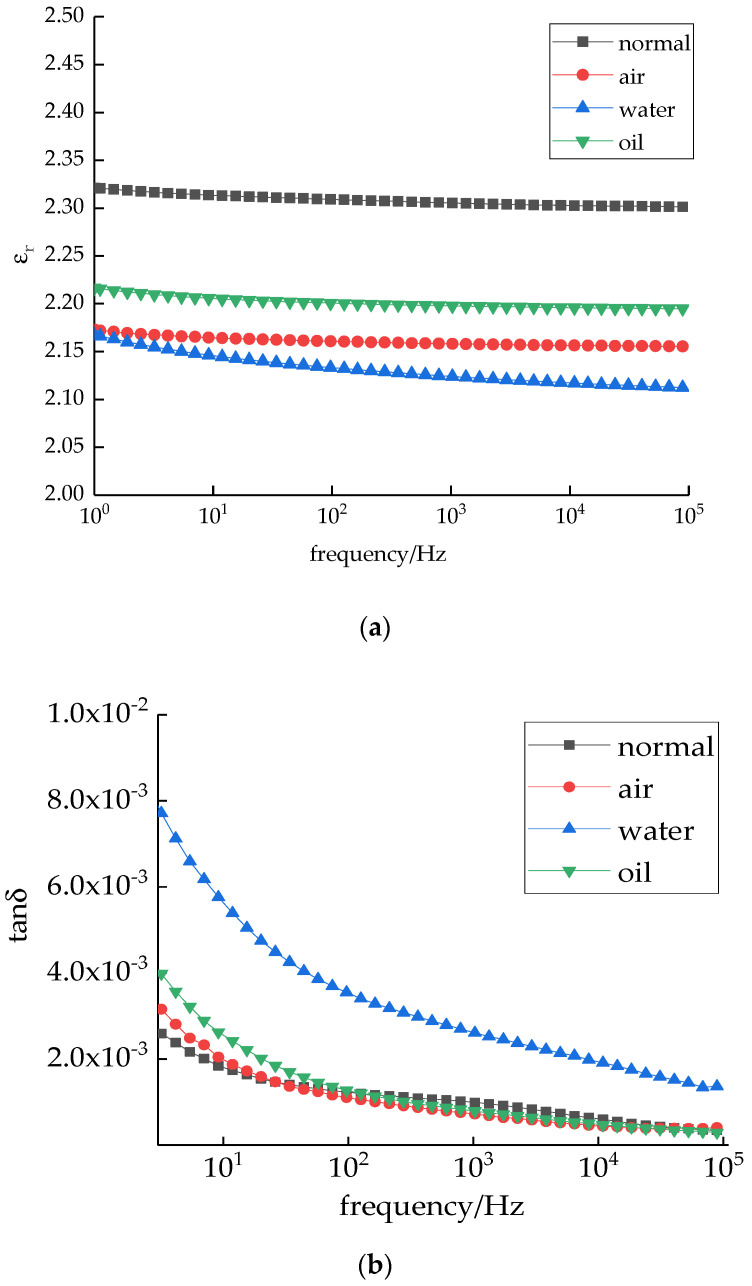
Dielectric spectrum test results of the different samples. (**a**) Variation of different sample dielectric constants with frequency. (**b**) Variation of different sample loss factors with frequency.

The different cooling media changed the microstructure of the LDPE, which affected the material polarization. Therefore, the dielectric constants and loss changed. In this study, a broadband dielectric spectroscopy analyzer was used to test the dielectric spectra of the different samples. The sample thickness was approximately 100 μm, and the diameter was approximately 30 mm. The two-electrode system was evaporated under vacuum. The experimental frequency range was 1–10^5^ Hz. The dielectric spectrum test results of the different samples are shown in Figure 10.

The relationship between the dielectric constants of different samples with frequency is shown in Figure 10a. With increasing frequency, the relative dielectric constants of all the test samples decreased slightly. This is because the polarization had sufficient time to be completed. Thus, the polarization was fully established. The relative dielectric constant was higher. Under a high frequency (higher than 10^5^ Hz), with an increasing frequency, small amounts of polarization cannot keep up with external electric field changes. Thus, polarization is not established. The relative dielectric constants decreased slightly. Among them, the relative dielectric constants of sample 1 were the highest, whereas the relative dielectric constants of samples 2 and 3 were lower. This is because the different cooling media changed the crystalline morphology of LDPE. The grain size decreased and the structure was closed, which limits the movement of the LDPE molecular segment. The dielectric constants decreased. The dielectric constant of sample 4 was higher than that of sample 3. This is because several impurities were introduced during the cooling process. Impurity polarization under an electric field increases the dielectric constant. The variation in different sample loss factors with frequency is shown in Figure 10b. The order of the four sample loss factors was as follows: sample 3 > sample 4 > sample 2 > sample 1. This is because impurities were introduced during sample preparation. The impurity relaxation polarization loss increased, which increased the loss factor. The loss of sample 3 was higher than that of the other three samples. This is because a water layer formed on the sample surface during the cooling process, which increased the surface conductive loss. In addition, water molecules entered the samples. Therefore, dipole polarization formed, which increased the loss.

## 5. Conclusions

In this study, a vulcanizing press was used for the melting press. Different LDPE film samples were prepared by combining different media for cooling crystallization. Based on the test results and analysis, the conclusions are as follows:

(1) The cooling medium has a significant effect on material crystallization, which decreases the grain size. The order of the four sample grain sizes was as follows: sample 1 > sample 2 > sample 3 > sample 4.
(2) The cooling media significantly affect the space charge accumulation and decay of LDPE. The injected space charges decreased, and thus, the remaining space charges were less. Under a 40 kV/mm field strength, the order of mean charge density of the four samples was as follows: sample 1 > sample 2 > sample 3 > sample 4. Under a short circuit, the mean charge density and mean charge decay rate of the different samples were similar. The order was as follows: sample 1 > sample 2 > sample 3 > sample 4. From the space charge test, after applying field strength for 20 min, the space charge density of sample 4 was the lowest. After a short circuit for 30 min, the residual space charge in sample 4 was the least. This illustrates that sample 4 could restrain space charges well.
(3) Under a low field strength or high field strength, the conductivity variations of the four test samples were different. Under a low field strength, the order of sample conductivity was as follows: sample 4 > sample 3 > sample 2 > sample 1. Under a high field strength, the order of sample conductivity was as follows: sample 2 > sample 1 > sample 4 > sample 3. In addition, the dielectric constant of sample 1 was higher, and the loss angular tangent value was lower.

## Figures and Tables

**Table 1:** Sample number, thickness, and cooling medium.

Sample Number	Cooling Methods	Thickness (μm)
1	Natural air cooling	100/200/300
2	Air rapid cooling	100/200/300
3	Water cooling	100/200/300
4	Oil cooling	100/200/300

**Table 2:** Space charge peak of different samples.

Samples	Charges Peak Value Around Negative Electrode		Charges Peak Value in Samples		Charges Peak Value around Positive Electrode	
	0.2 min	300 min	0.2 min	300 min	0.2 min	300 min
Sample 1	2.03968	0.8799	1.42489	0.57825	1.90079	0.70351
Sample 2	1.9548	0.91369	0.90586	0.40603	2.42349	0.78021
Sample 3	1.50794	0.76642	0.54015	0.18552	0.9764	0.4663
Sample 4	1.44536	0.80504	0.31285	0.16637	0.64648	0.24805
